# Phosphotungstic Acid: An Efficient, Cost-effective and Recyclable Catalyst for the Synthesis of Polysubstituted Quinolines

**DOI:** 10.3390/molecules14031126

**Published:** 2009-03-12

**Authors:** Minoo Dabiri, Sahareh Bashiribod

**Affiliations:** 1 Department of Chemistry, Faculty of Science, Shahid Beheshti University, G. C., Evin 1983963113, Tehran, Iran; 2 Department of Marine Biology, Faculty of Biological Scinences Shahid Beheshti University, G. C., Evin, Tehran, Iran

**Keywords:** Heteropolyacid, Heterogeneous catalysis, Friedländer annelation, Quinoline.

## Abstract

Phosphotungstic acid (H_3_PW_12_O_40_) was used as an efficient and recyclable catalyst for the synthesis of polysubstituted quinolines through the Friedländer condensation of 2-aminoarylketone with carbonyl compounds, which was achieved by conventional heating under solvent-free conditions.

## Introduction

Over the past 20 years, the chemistry community, and in particular, the chemical industry, has made extensive efforts to reduce the risks associated with the manufacture and use of various chemicals. Green chemistry is an approach to the synthesis, processing, and use of chemicals that aims to reduce the risks to humans and the environment. Much innovative chemistry has been developed over the past several years that is effective, efficient and more environmentally benign.

In recent years, the use of solid acids as heterogeneous catalysts has received considerable attention in different areas of organic synthesis [1]. Amongst the various heterogeneous catalysts, heteropolyacids (HPAs) are some of the most attractive, because they are commercially available, easy to handle, they display remarkably low toxicity, are environmentally friendly, economically cost effective, they possess very high Brønsted acidity, they constitute a mobile ionic structure and absorb polar molecules easily in the bulk, forming a ‘pseudoliquid phase’ [2,3]. As a result, both the surface protons and the bulk protons of HPAs participate in their catalytic activity, which significantly enhances the reaction rate. The best known HPAs are the Keggin HPAs, H_8_n_XM_12_O_40_, where X is the central atom (Si^4+^, P^5+^, etc.), n is the oxidation state of X and M is the metal ion (W^6+^ or Mo^6+^). Of these, phosphomolybdic acid, phosphotungstic acid and silicotungstic acid, in particular, have been used in recent years for the synthesis of various heterocycles [4,5,6,7,8].

Quinolines are very important compounds because of their pharmacological properties. Members of this family have wide applications in medicinal chemistry, being used as antimalarial, anti-inflammatory, antiasthamatic, antibacterial, antihypertensive, and tyrosine kinase inhibiting agents [9,10,11]. The structural core of quinoline has generally been synthesized by various conventional named reactions [12]. Among them, Friedländer annelation is the most simple and straightforward method for the synthesis of polysubstituted quinolines. The Friedländer synthesis involves an acid or base catalyzed condensation between 2-aminoaryl ketone and a second carbonyl compound containing a reactive α-methylene group followed by a cyclodehydration. Brønsted acids and Lewis acids are known to promote these reactions [13,14,15,16,17,18,19]. Brønsted acid catalysts, such as hydrochloric acid [20], perchloric acid [15], sulfamic acid [21], oxalic acid [22], silver phosphotungstate [23] and NiCl_2_·2H_2_O [24] have been used in Friedländer reactions. However, in spite of their potential utility, some of these catalysts present limitations due to the use of toxic and corrosive reagents, the tedious workup procedures, the necessity of neutralization of the strong acid media, producing undesired washes, long reactions times, and high temperatures. Moreover, the synthesis of these heterocycles has been usually carried out in a solvent such as THF, DMF, or DMSO leading to complex isolation and recovery procedures. Therefore, the introduction of a novel and inexpensive heterogenous catalyst, which can be easily separated, reused, and does not become contaminated by the products, is of prime importance.

## Results and Discussion

In continuation of our ongoing interest in solvent-free synthesis [25,26,27,28,29,30], we herein report the use of phosphotungstic acid (H_3_PW) [3] as a catalyst in the synthesis of quinolines with excellent yields by the reaction of a variety of α-methyleneketones and 2-aminoaryl ketones under mild reaction conditions. 

**Scheme 1:**
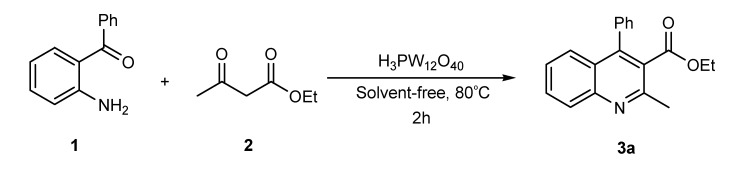
The reaction of 2-aminobenzophenone (**1**), and ethyl acetoacetate (**2**) under solvent-free condition at 80 ºC in the presence of phosphotungstic acid.

Our initial investigation was focused on the use of H_3_PW as the catalyst in the reaction of 2-amino-benzophenone (1), and ethyl acetoacetate (2) under solvent-free condition at 80 ºC (Scheme 1). It is worthy to note that in all reactions, the conditions were optimized for a 100% conversion. As could be seen in Table 1, the best result was obtained in the presence of 1 mol% of H_3_PW and any further increase n the reaction time did not have any effect on the yield. Furthermore, we also tested the catalytic activity of different catalysts such as HClO_4_, *p*-toluenesulfonic acid (T*s*OH), H_2_SO_4_, silica sulfuric acid (SSA), and ZnCl_2_, and obtained only moderate yields under solvent-free conditions. One of the most interesting points in this work is the difference of the catalytic activity between simple mineral acids (HClO_4_ and H_2_SO_4_, Table 1, entries 13, 14) and H_3_PW under solvent-free condition (Table 1). Encouraged by this result, we turned our attention to various substituted substrates. The procedure gave the products in high yields and avoids problems associated with solvents and liquid acids use (cost, handling, safety, pollution, corrosiveness, separation, and recovery) (Table 2). In a control experiment, it was observed that in the absence of the catalyst, the reaction did not proceed even at higher temperatures. Lowering the reaction temperature was detrimental to the efficiency of this procedure.

**Table 1:** Effect of amounts of H_3_PW_12_O_40_ and other catalysts on the synthesis of **3a** by the reaction of 2-aminobenzophenone (**1**), and ethyl acetoacetate (**2**).

Entry^a^	Catalyst (mol%)	Yield (%)^b^	Time (h)
1	H_3_PW_12_O_40 _(0.1)	30	24
2	H_3_PW_12_O_40 _(0.2)	45	20
3	H_3_PW_12_O_40 _(0.3)	50	12
4	H_3_PW_12_O_40 _(0.4)	50	8
5	H_3_PW_12_O_40 _(0.5)	50	6
6	H_3_PW_12_O_40 _(0.6)	55	5
7	H_3_PW_12_O_40 _(0.7)	60	3.5
8	H_3_PW_12_O_40 _(0.8)	75	3
9	H_3_PW_12_O_40 _(0.9)	83	2.5
10	H_3_PW_12_O_40 _(1)	90	2
11	H_3_PW_12_O_40 _(1.1)	91	2
12	H_3_PW_12_O_40 _(1.2)	91	2
13	HClO_4_ (1)	40	12
14	H_2_SO_4_ (1)	30	12
15	SSA (1)	60	5
16	ZnCl_2_ (1)	40	12
17	TsOH (1)	50	12

^a ^In all reaction the conditions were optimized for a 100% conversion; ^b ^Isolated yield.

**Table 2:** Synthesis of quinolines in the presence of H_3_PW_12_O_40_ under solvent-free conditions at 80˚C in 2 hours.

Entry	2-Aminoaryl ketone	CH-acid	Product	Yield (%)^a^	Mp (˚C) Found	Mp (˚C) Reported
1	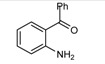	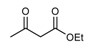	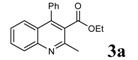	90	102-103	100-101^b^
2	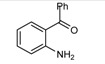	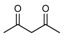	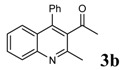	92	110-112	111-112^b^
3	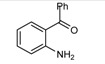	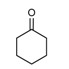	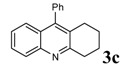	94	153-154	156-157^c^
4	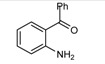	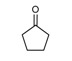	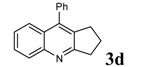	90	130-131	130-132^c^
5	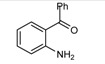	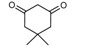	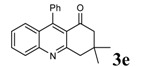	89	192-194	190-192^c^
6	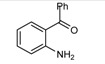	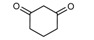	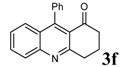	92	155-157	155-156^c^
7	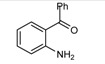	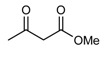	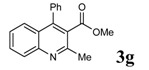	93	99-100	98-100^d^
8	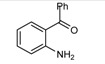	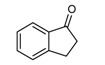	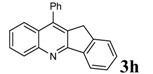	95	152-154	153-154^e^
9	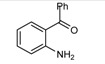	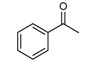	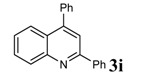	94	103-105	105-107^f^
10	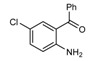	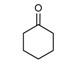	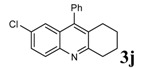	95	164-166	164-165^c^
11	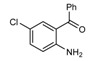	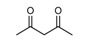	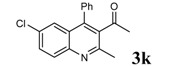	91	150-152	150-151^c^
12	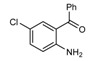	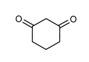	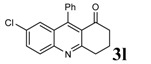	89	186-187	185-186^c^
13	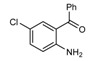	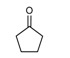	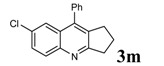	88	106-108	106-107^b^
14	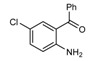	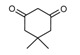	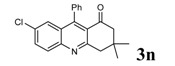	92	207-209	208-209^c^
15	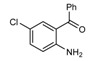	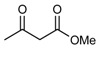	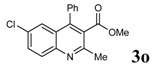	94	132-134	133-135^b^

^a^Isolated yield,^ b^Reference [22],^ c^Reference [23],^ d^Reference [31],^ e^Reference [32] and^ f^Reference [33].

In acid-catalyzed reactions by heteropolyacids, several types of acid sites are present [2,3]. They include proton sites in bulk heteropolyacids, Lewis acid sites in their salts (metal counterions), proton sites in acidic salts, proton sites generated by dissociation of coordinated water and reduction of salts, and proton generated by partial hydrolysis of polyanions. Generally, reactions catalyzed by heteropolyacids may be represented by the conventional mechanisms of Brønsted acid catalysis. The mechanism may include the protonation of the substrate followed by the conversion of the ionic intermediate to yield the reaction product [2,3].

The catalyst, *i.e.* H_3_PW_12_O_40_, was recovered by simple filtration and reused in subsequent runs with no decrease in activity. The possibility of recycling the catalyst is one of the key advantages of this procedure, which was demonstrated using 2-aminobenzophenone (1), and ethyl acetoacetate (2) as a model reaction. At the end of the reaction, the reaction mixture was washed with water, dried at 130 ˚C for 1 h, and the catalyst was reused in another reaction. We have found that the catalyst could be reused several times without any appreciable loss of activity (Table 3).

**Table 3:** Reusability of H_3_PW in the reaction of 2-amino benzophenone (**1**), and ethyl acetoacetate (**2**) under solvent-free conditions.

Run No.	Yield (%)^a^	Time (h)
1	93	2
2	93	2
3	92	2.5
4	91	2.5
5	92	3

^a ^Isolated yield based on 2-aminobenzophenone.

In conclusion, a simple, convenient and efficient protocol for the synthesis of wide range of quinolines under solvent-free conditions is reported. The high yields of products, easy work up procedure, and use of a very small amount of heteropolyacid make it the preferred procedure for the preparation of different kind of quinolines. Another important feature of this methodology is the use of heteropoly acid as catalyst, and avoidance of hazardous organic solvent.

## Experimental

### General

Melting points were measured on an Electrothermal 9200 apparatus and are not corrected. Mass spectra were recorded on a FINNIGAN- MAT 8430 mass spectrometer operating at an ionization potential of 70 eV. ^1^H- and ^13^C-NMR spectra were recorded on a Bruker DRX-300 AVANCE spectrometer at 300.13 and 75.47 MHz, respectively, using DMSO-*d_6_* as the solvent. All chemical reagents were obtained from Fluka and Merck chemical companies and were used without purification.

### General procedure for the synthesis of quinolines in the presence of heteropolyacid

A mixture of 2-aminoarylketone (1.0 mmol), α-methyleneketones (1.2 mmol) and phosphotungstic acid (0.04 g, 0.01 mmol) was mixed thoroughly and heated under solvent-free conditions at 80 ºC. After completion of the reaction, as indicated by TLC (eluent: 2:1 *n*-hexane/ethyl acetate), the reaction mixture was washed with water, because phosphotungstic acid is soluble in water. Then the solid product was filtered off and recrystallised from ethanol.

### Spectral data for **3g**, **3h** and **3i**

*Methyl 2-methyl-4-phenylquinoline-3-carboxylate* (3g): White solid; Mp 99-100 °C; IR (KBr) (ν_max_, cm^-1^): 1731 (C=O), 1229, 761, 3433; ^1^H-NMR δ = (ppm) 2.66 (s, 3H, CH_3_), 3.54 (s, 3H, CH_3_), 7.32-8.05 ( m, 9H); ^13^C-NMR δ = 23.7 (CH_3_), 52.6 (CH_3_), 124.7, 126.4, 127.3, 127.4, 128.8, 129.1, 129.4, 131.0, 135.3, 145.9, 147.6, 154.2, 168.6 (C=O); MS (EI, 70 eV): m/z (%): 277 (M^+^, 100).

*10-Phenyl-11H-indeno*[1,2-b]*quinoline* (3h): Yellow solid; Mp 152-154 °C; IR (KBr) (ν_max_, cm^-1^): 724, 765, 3423; ^1^H-NMR δ = (ppm) 3.81 (s, 2H, CH_2_), 7.47-7.76 (m, 13H), 8.14-8.2 (m, 2H); ^13^C- NMR δ = 19.0, 34.1, 56.5, 122.3, 125.9, 126.0, 126.2, 126.8, 127.9, 129.0, 129.2, 129.7, 129.9, 131.1, 133.5, 135.7, 138.9, 144.7, 146.2, 146.5, 159.8; MS (EI, 70 eV): m/z (%): 293 (M^+^, 100).

*2,4-Diphenylquinoline* (3i): White solid; Mp 103-105 °C; IR (KBr) (ν_max_, cm^-1^): 696, 767, 3437; ^1^H- NMR δ = (ppm) 7.47-7.58 (m, 9H), 7.73-7.83 (m, 2H), 7.97 (s, 1H), 8.14-8.31 (m, 3H); ^13^C-NMR δ = 119.2, 125.5, 125.6, 127.1, 127.8, 129.0, 129.1, 129.2, 130.0, 130.04, 130.2, 130.23, 138.0, 139.0, 148.6, 149.0, 156.1; MS ( EI, 70 eV): m/z (%): 281 (M^+^, 100).
